# Gestational Diabetes Mellitus: New Thinking on Diagnostic Criteria

**DOI:** 10.3390/life14121665

**Published:** 2024-12-16

**Authors:** Jiyu Luo, Ling Tong, Ao Xu, Yihan He, Haiyun Huang, Dongmei Qiu, Xiaoyu Guo, Hongli Chen, Lingyun Xu, Yang Li, Hongling Zhang, Yuanyuan Li

**Affiliations:** 1 School of Medicine and Health, Wuhan Polytechnic University, Wuhan 430023, China; jiyuluo0615@outlook.com (J.L.); aoxu0624@outlook.com (A.X.); heyihanha@foxmail.com (Y.H.); amyhuang9912@gmail.com (H.H.); qdm1232023@163.com (D.Q.); 2 School of Health and Nursing, Wuchang University of Technology, Wuhan 430223, China; 120130016@wut.edu.cn; 3 School of Life Science and Technology, Wuhan Polytechnic University, Wuhan 430023, China; 17683931781@163.com (X.G.); 18827021016@163.com (H.C.); doctorxly9898@163.com (L.X.); yangli@whpu.edu.cn (Y.L.); 4 Key Laboratory of Environment and Health, Ministry of Education & Ministry of Environmental Protection, and State Key Laboratory of Environmental Health (Incubation), School of Public Health, Tongji Medical College, Huazhong University of Science and Technology, Wuhan 430030, China

**Keywords:** gestational diabetes mellitus, diagnostic criteria and method, IADPSG, screening, oral glucose tolerance test

## Abstract

Currently, there is a lack of standardized diagnostic criteria for gestational diabetes mellitus (GDM), making it a subject of ongoing debate. The optimal diagnostic method and screening strategy for GDM remain contentious. In this review, we summarize the criteria and methods for diagnosing GDM, and perform a comparison between universal and selective screening strategies. Therefore, this review aims to highlight the following: (1) The most widely adopted criteria for GDM are those established by the International Association of Diabetes and Pregnancy Study Groups (IADPSG). (2) Evidence from cohort studies suggests that the one-step diagnostic method is associated with improved pregnancy outcomes and appears more cost-effective compared to the two-step method. (3) Universal screening is more cost-effective than selective screening, which may overlook a significant number of women with GDM. Additionally, various methods have been proposed for early pregnancy screening (before 14 weeks). Finally, an outlook is presented for the diagnosis of GDM, emphasizing the importance of large-scale randomized controlled trials (RCTs) to provide stronger evidence for future support.

## 1. Introduction

GDM is defined as any degree of glucose intolerance with onset or initial recognition during pregnancy [1]. In 2022, the estimated prevalence of gestational diabetes worldwide was 14% [2], with a projected rate of 11% in China [3]. This prevalence has shown a consistent year-on-year increase. Pregnancy-related hyperglycemia is linked to a series of adverse pregnancy outcomes for mothers and infants. Women with GDM face an increased risk of complications such as gestational hypertension, polyhydramnios, preeclampsia, and cesarean delivery [4,5,6,7]. Additionally, the risk of developing type 2 diabetes mellitus (T2DM) in women with GDM is sevenfold that of women with normal glucose tolerance during pregnancy. The offspring born to mothers with GDM are at an elevated risk of straightforward complications, including macrosomia, shoulder dystocia, birth injury, preterm birth, neonatal hypoglycemia, neonatal unit admission, and respiratory distress [4,5,6,7,8]. These children also face increased risks of obesity, insulin resistance, systolic blood pressure, and circulatory diseases later in life [9,10]. Therefore, it is of great importance to accurately screen and diagnose pregnant women with GDM with the aim of declining the incidence of adverse perinatal and long-term outcomes. However, no global consensus exists regarding the timing of screening, diagnostic tests, and thresholds for GDM. To date, the one- or two-step method for the diagnosis of GDM is recommended by many professional associations. The most utilized criteria are shown in Table 1.

## 2. Evolution of the Diagnostic Criteria for GDM

**Figure 1:**
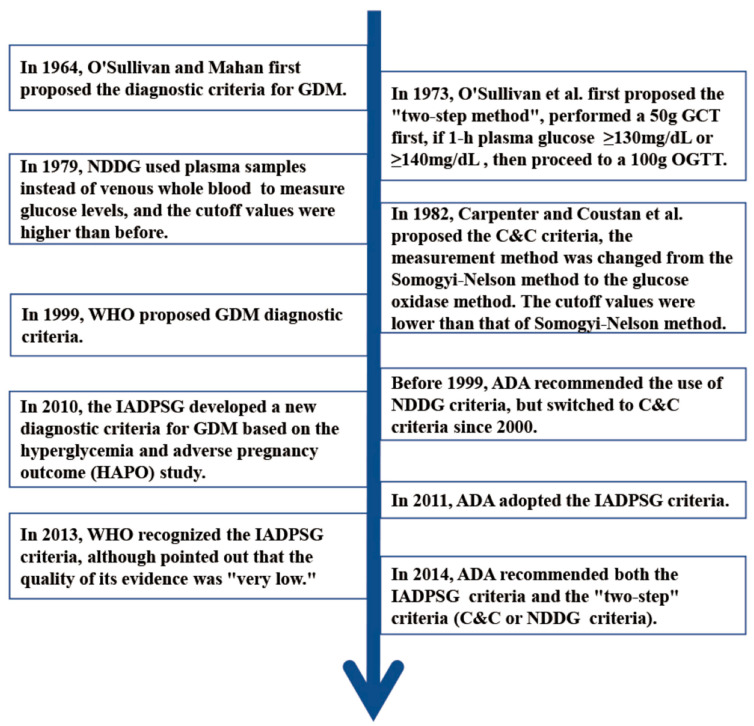
Evolution of the diagnostic criteria for GDM [O’Sullivan and Mahan+1964; O’Sullivan+1973; NDDG+1979; Carpenter and Coustan+1982; WHO+1999; ADA+2000; IADPSG+2010; ADA+2011; WHO+2013; ADA+2014].

The diagnostic criteria for GDM have evolved significantly over time. In 1964, O’Sullivan and Mahan proposed a novel diagnostic criterion for GDM [11]. A 3 h 100 g oral glucose tolerance test (OGTT) was employed to diagnose hyperglycemia in pregnant women. They randomly selected 732 pregnant women to take 100 g glucose orally and used the Somogyi–Nelson method to measure venous whole-blood glucose values, including fasting, 1 h, 2 h, and 3 h values after taking glucose. When at least two glucose values obtained during the OGTT exceeded the mean value by two standard deviations or more, then a diagnosis of GDM was made. The original diagnostic cut-off values were 90, 165, 145, and 125 mg/dL (5.0, 9.2, 8.0, 6.9 mmol/L). The criterion was proposed based on the risk of T2DM in pregnant women after delivery. In 1973, O’Sullivan proposed the two-step method. First, a 50 g glucose challenge test (GCT) was conducted. Then if the 1 h glucose level exceeded 140 mg/dL (7.8 mmol/L), a follow-up 100 g OGTT was conducted. They continued to the OGTT if the pregnant women did not pass the GCT [18]. The diagnosis criteria for the subsequent decades came from the evolution of the O’Sullivan criteria (see Figure 1).

In 1979 [12], the National Diabetes Data Group (NDDG) revised the thresholds, increasing each value by approximately 15% to reflect the shift from whole-blood to plasma glucose measurement. The updated diagnostic cut-off values were 105, 190, 165, and 145 mg/dL (5.8, 10.6, 9.2, and 8.1 mmol/L). In 1982 [13], based on a novel glucose oxidase approach for plasma glucose measurement, the Carpenter–Coustan (C&C) criteria initially came off the press. The criteria were designed to substitute the conventional Somogyi–Nelson technique. The latter was susceptible to interference from several non-glucose substances. The cut-off values were modified to 95, 180, 155, and 140 mg/dL (5.3, 10.0, 8.6, 7.8 mmol/L), which were lower than those of the Somogyi–Nelson method. Prior to 1999, the American Diabetes Association (ADA) had suggested the use of the NDDG criteria to diagnose GDM. However, in 2000, the ADA switched to recommending the C&C criteria. It is noteworthy that the NDDG and C&C criteria were founded on a 100 g 3 h OGTT following a 50 g GCT with a cut-off value ranging from 130 to 139 mg/dL (7.2 to 7.7 mmol/L). The ADA endorsed this combination of the two-step diagnostic methods prior to 2010. In 2010, the IADPSG introduced new criteria based on maternal and neonatal outcomes. The fasting plasma glucose (FPG), 1 h and 2 h blood glucose cut points after OGTT of IADPSG were lower than other GDM standards, which had positive significance for improving adverse pregnancy outcomes. Since 2011, the ADA has endorsed the International Association of Diabetes and Pregnancy Study Groups (IADPSG) criteria, which suggest the one-step method employing the 75 g 2 h OGTT with one or more abnormal values [19]. However, the ADA revised its proposals to support two-step methods (NDDG or C&C) and the IADPSG criteria in 2014 [20].

In contrast, the ADA, which specifically formulated diagnostic criteria for GDM in pregnant women, recommended the World Health Organization (WHO) criteria in 1999. These criteria advised that GDM should be diagnosed in accordance with the standards criteria as diabetes mellitus or impaired glucose tolerance in the general population. After a 75 g 2 h OGTT, if the FPG ≥ 126 mg/dL (7.0 mmol/L) or 2 h plasma glucose ≥ 140 mg/dL (7.8 mmol/L), GDM could be diagnosed with one or more abnormal values. In comparison to previous guidelines, the abnormal cut-off values have declined [15]. In 2013, the WHO adopted the IADPSG criteria but acknowledged the limited quality of evidence supporting them.

In summary, the diagnostic criteria for GDM have undergone numerous changes, including shifts from whole-blood to plasma glucose measurements, transitions from the Somogyi–Nelson method to glucose oxidase methods, modifications from the one-step to two-step method, changes from the 100 g OGTT to the application of 75 g OGTT, and adjustments in the abnormal values from two to one. In the end, the formulation of criteria has been based on a series of considerations, including the risks of T2DM in pregnant women and the impact on pregnancy outcomes.

## 3. The Hyperglycemia and Adverse Pregnancy Outcomes (HAPO) Study

Before the HAPO study, it was widely recognized that overt diabetes mellitus significantly increased the risk of adverse perinatal outcomes during pregnancy. Nevertheless, whether maternal hyperglycemia, less severe than overt diabetes, also elevated these risks remained a contentious issue. In order to address this question, the HAPO examined a diverse cohort comprising individuals from a range of ethnic backgrounds and countries of origin. The HAPO study was conducted by the National Institutes of Health (NIH) in 2000. It was a multicenter, prospective study involving over 25,000 pregnant women from 15 medical centers across nine countries, including sites in North America, Europe, Asia, the Middle East, and Australia. In Asia, research was conducted at hospitals in Bangkok, Thailand, Hong Kong, and Singapore. In the HAPO study, GDM was diagnosed by using a 75 g OGTT between 24 and 32 weeks of gestation, measuring the FPG, 1 h, and 2 h plasma glucose levels.

The primary concern for obstetricians was related to the severity and frequency of adverse pregnancy outcomes, rather than the long-term risk of maternal diabetes. Prior to the HAPO, studies demonstrated that undiagnosed GDM was linked to an elevated risk of perinatal mortality [21,22]. However, in many study sites, perinatal mortality rates were particularly low, making it exceedingly challenging to identify this as one of the adverse pregnancy outcomes. Consequently, the HAPO study focused on morbidities directly closely correlated to maternal metabolic disturbances of diabetes mellitus. Primary outcomes of the HAPO study included birth weight > 90th percentile, cesarean section delivery, clinically defined neonatal hypoglycemia, and fetal hyperinsulinemia (cord C-peptide > 90th percentile). Secondary outcomes encompassed preeclampsia, preterm delivery, shoulder dystocia/birth injury, hyperbilirubinemia, and intensive neonatal care.

The HAPO study demonstrated significant, continuous associations between rising levels of fasting, 1 h, and 2 h plasma glucose which were acquired by OGTT and primary outcomes, including macrosomia (birth weight > 90th centile) and fetal hyperinsulinemia (cord C-peptide > 90th centile) [7]. Associations with cesarean delivery and neonatal hypoglycemia were weaker. Additionally, the study revealed a positive correlation between elevated plasma glucose levels during pregnancy and a rising risk of five secondary outcomes, including preterm delivery, preeclampsia, intensive neonatal care, birth injury, hyperbilirubinemia, and shoulder dystocia. This cohort study was designed to elucidate the correlation between pregnant glucose levels below previous diagnostic criteria and adverse pregnancy outcomes. However, no discernible inflection points were identified.

In recent years, attention has shifted toward the long-term consequences of GDM for mothers and children. Moreover, the HAPO research group also conducted the HAPO Follow-up Study (HAPOFUS) to determine the correlation between maternal glycemia during pregnancy and childhood glucose metabolism in the cohort [23,24]. The HAPOFUS included more than 4000 children aged 10–14 years who completed all or part of an OGTT, and whose mothers underwent a 75 g OGTT at 24–28 weeks of gestation. The primary outcomes for children were impaired fasting glucose (IFG) and impaired glucose tolerance (IGT). Additional outcomes included glycated hemoglobin (HbA1c) and C-peptide, among others. The HAPOFUS findings revealed a significant correlation between maternal hyperglycemia and childhood glucose levels and insulin resistance, which was independent of family history of diabetes and childhood BMI.

## 4. The International Association of Diabetes in Pregnancy Study Groups (IADPSG) Criteria

Following the HAPO study findings, the IADPSG recommended a one-step 75 g OGTT diagnostic criterion in 2010. This criterion stipulates that at least one glucose value must reach or exceed the specific cut-offs [16]. The findings of the HAPO study showed a lack of inflection points in the correlation between maternal glucose levels and adverse pregnancy outcomes. Considering this, the IADPSG convened a consensus group meeting to determine appropriate thresholds. Furthermore, the possibility of utilizing an OGTT cut-off that recognized odds ratios (ORs) of 1.5, 1.75, or 2.0 (in comparison to mean values) for the risk of fetal macrosomia, neonatal adiposity, and fetal hyperinsulinemia (all defined as above the 90th percentile) was considered. Following a vote, the Consensus Panel determined that an odds ratio of 1.75 would be an appropriate selection for these outcomes, relative to the average glucose values for the entire study cohort. This corresponded to glucose levels of fasting ≥ 92 mg/dL (5.1 mmol/L), 1 h ≥ 180 mg/dL (10.0 mmol/L), and 2 h ≥ 153 mg/dL (8.5 mmol/L) on a 75 g OGTT.

Despite notable discrepancies in the prevalence of GDM across the 15 study centers when utilizing the IADPSG criteria, the correlation between glucose and prevention remained consistent. Consequently, it was deemed appropriate to implement the criteria globally, encompassing countries and regions that were not included in the HAPO study.

A lot of international institutions adopted the IADPSG criteria later. In 2011, the ADA recommended the IADPSG criteria [25]. The WHO redefined GDM and recommended the criteria in 2013, although the quality of its evidence was considered “very low” [26]. Moreover, the IADPSG criteria were recognized by the International Federation of Gynecology and Obstetrics (FIGO) [27]. In December 2011, the Chinese Ministry of Health (MOH) issued a set of guidelines recommending the use of the IADPSG criteria [28].

## 5. Impacts of Application of the IADPSG Criteria

The most significant influence of adopting the IADPSG criteria is the considerable rise in the incidence of GDM. To evaluate the current strategies for screening and diagnosis, the European Board and College of Obstetrics and Gynecology (EBCOG) conducted an online survey. This included 28 European countries for diagnosis of GDM from September to November 2015. The outcomes of the survey indicated that the IADPSG criteria were the most frequently utilized diagnostic criteria for GDM, accounting for 67.9% of cases [29]. This was followed by the 1999 WHO criteria (10.7%), the European Association for the Study of Diabetes criteria (7.1%), and the C&C criteria (7.1%). Countries that did not intend to adopt the IADPSG criteria (such as the United Kingdom) believe that there was insufficient evidence to implement the IADPSG criteria and it would lead to a significant rise in the prevalence of GDM and costs.

A Norwegian cohort study compared GDM prevalence using the 1999 WHO criteria and the IADPSG criteria. It found that the prevalence of GDM was 2.4 times higher under the IADPSG criteria [30]. Similarly, several scientific societies observed that adopting the IADPSG criteria significantly increased GDM prevalence [31]. A Canadian cohort study compared the IADPSG criteria and the Canadian Diabetes Association (CDA) criteria [32]. The results demonstrated that GDM prevalence rose from 3.2% to 10.3% under the IADPSG criteria.

The primary reason for the increase was the lower diagnostic thresholds of the IADPSG criteria, where a diagnosis requires only one abnormal value instead of two. Notably, using a single abnormal value contributed to a 5.3% increase in GDM rates, while lower threshold values accounted for a 1.8% rise. Among the three threshold values, the 1 h threshold was the most significant factor driving the higher GDM rate. This observation aligns with findings published in the *British Medical Journal* [33].

## 6. Current Disputes over the Application of Diagnostic Criteria

The IADPSG criteria have gained widespread acceptance over the past few decades. Although the one-step method offers certain advantages [34,35], it also increases GDM prevalence [34,35,36] and places additional strain on healthcare systems [34,37,38,39]. Debate continues over the most effective diagnostic strategy, with particular focus on the following aspects:

(1) Could the IADPSG criteria be used globally?
(2) Choose 1. 75 or 2. 0 for odds ratio value?
(3) One-step versus two-step diagnostic method.
(4) Universal screening versus selective screening.
(5) Early screening for overt diabetes and GDM.

### 6.1. Could the IADPSG Criteria Be Used Globally?

Although the IADPSG criteria are applied globally, significant variability exists across centers in the frequency of GDM diagnoses and the diagnostic relevance of fasting, 1 h, and 2 h glucose levels. These discrepancies influence the strategies used for diagnosing GDM. In a study by Sacks et al. [36], part of the HAPO Study, the prevalence of GDM across 15 centers using the IADPSG criteria ranged from 9.3% to 25.5%, with an overall prevalence of 17.8%. This diversity could be attributed to differences in ethnic origin and age difference between the study populations, and the global epidemic of obesity and diabetes [40].

**Figure 2:**
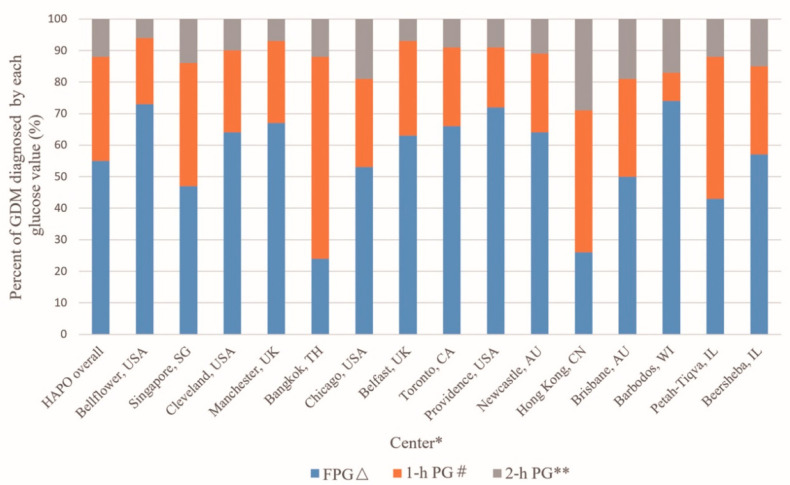
Frequency of GDM and diagnostic proportion of individual glucose value among 15 centers in the HAPO study. [* Centers listed from highest to lowest unadjusted frequency of GDM; Δ: Includes all with FPG ≥ threshold without regard to 1 h and 2 h value; #: Includes all with FPG < threshold and 1-h ≥ threshold without regard to 2-h value. ** Only 2 h value ≥ threshold.]

Furthermore, the HAPO study also highlighted substantial differences in the diagnostic contributions of the FPG, 1 h, and 2 h glucose levels. Figure 2 illustrates the percentage of GDM which is diagnosed by each glucose measure while fasting, 1 h, and 2 h glucose values. Overall, a 55% FPG glucose value met the cut-off for GDM, and the 1 h value had 33% diagnostic value, but the 2 h value only had 12% diagnostic value. FPG accounted for 24% and 26% of GDM diagnoses in Hong Kong and Bangkok, respectively. However, in Barbados, Bellflower, and Providence, it accounted for over 70%. Similarly, the diagnostic weight of the 1 h OGTT value varied significantly, ranging from 9% in Barbados to 64% in Bangkok. In contrast, the 2 h OGTT value showed less variability, with contributions between 6% in Bellflower and 29% in Hong Kong. Therefore, these findings raise questions about whether a unified diagnostic criterion is appropriate, given racial and regional differences. In addition, the HAPO study population did not include participants from India or several other Asian countries, highlighting a gap in data and the need to assess the long-term applicability of the IADPSG criteria in Asia. Differences in GDM frequency and the diagnostic weight of a single glucose value across different regions may impact the selection of GDM diagnostic strategies in different regions or populations and bring difficulties during the implementation of uniform criteria worldwide. For instance, within a population where more than half of the women with GDM meet the FPG diagnostic threshold, it is necessary to implement precise FPG measurement as an initial step and reserve a comprehensive OGTT for those who do not reach the threshold. Similarly, in Bellflower, Manchester, and Providence, the diagnostic weight of the 2 h OGTT value is less than 10%. This suggests that it may be reasonable to implement only a 1 h OGTT. However, this strategy is not suitable in Hong Kong, because among patients in Hong Kong, the diagnostic weight of the 2 h OGTT value could reach 29%. Thus, regional variations necessitate flexible diagnostic criteria tailored to local populations to optimize GDM detection and management.

### 6.2. Choose 1.75 or 2.0 for Odds Ratio Value?

The IADPSG criteria established diagnostic thresholds based on an odds ratio (OR) of 1.75, a decision made through consensus rather than empirical evidence. This has sparked debate, particularly regarding whether expert opinion and experience can replace robust scientific evidence. A key point of contention is the absence of support from randomized clinical trials (RCTs) to validate these thresholds. A recent Danish cohort study by Mclntyre et al. [41] questioned the universal applicability of the IADPSG thresholds. In a population of northern European Caucasian, which was generally regarded as a low-risk group for GDM, with the use of the IADPSG threshold of FPG ≥ 92 mg/dL (5.1 mmol/L), 40% of the cohort could be diagnosed with GDM. Nevertheless, there was no evidence to demonstrate pregnancy hypertension or excessive fetal growth in this population until the FPG was ≥101 mg/dL (5.6 mmol/L), which was well above the IADPSG cut-off of 92 mg/dL (5.1 mmol/L). Additionally, no link was found between fasting glucose levels and cesarean section rates. The results were similar to a study in Spain [42], which reported a sharp rise in the large for gestational age (LGA) risk and gestational hypertensive disorders (GHDs) (including gestational hypertension and preeclampsia) when the FPG value was above 95–99 mg/dL (5.3–5.5 mmol/L). It was possible that the Danish population exhibited a distinctive profile, because the distribution of FPG levels in this Danish cohort was substantially higher than that in the HAPO cohort. Additionally, in this cohort, GDM prevalence exceeded 50% among women with a BMI ≥ 29, which is the highest rate reported in European studies [43].

Given these observations, the OR value of 1.75 chosen by the IADPSG warrants reassessment. If the OR value was set to 2.0, the diagnostic thresholds would be improved: FPG ≥ 95 mg/dL (5. 3 mmol/L), 1 h glucose ≥ 190 mg/dL (10.6 mmol/L), and 2 h glucose ≥ 162 mg/dL (9.0 mmol/L). These revised cut-offs would result in a notable reduction in the prevalence of GDM, and the FPG value would also align with the C&C criteria [44]. A retrospective cohort study assessed the impact of different glycemic thresholds (OR = 1.75 vs. OR = 2.0) on adverse pregnancy outcomes using the 75 g 2 h OGTT [45]. The glycemic thresholds were founded on the HAPO study outcomes (OR = 1.75 vs. OR = 2.0). It found that adopting the OR of 2.0 resulted in more adverse pregnancy outcomes compared to the IADPSG thresholds (OR = 1.75). Sacks et al. [45] emphasized the necessity for RCTs to ascertain the efficacy of treatment in women with mild GDM.

### 6.3. One-Step Versus Two-Step Diagnostic Method

Another controversy is about using the one- or two-step diagnostic method (see Table 2). The one-step method compared to the two is more sensitive and can detect mild GDM cases. In a large randomized trial encompassing 23,792 pregnant women, Hillier et al. [46] revealed that the prevalence of GDM screened by the one-step method was approximately twice that screened by the two-step method. As a result, the one-step method generally diagnosed a greater number of women with GDM. However, it remains uncertain whether women diagnosed by the one-step method, using the IADPSG criteria but not the two-step method, face an increased risk of GDM complications compared to women without GDM. Caissutti et al. examined four control groups and five study groups using data from an electronic database involving 29,983 pregnant women [47]. Their findings indicated that women meeting the IADPSG criteria through the one-step method but not the stricter criteria (C&C or CDA criteria) under the two-step method still had a heightened risk of adverse pregnancy outcomes, including gestational hypertension, preeclampsia, and large for gestational age risk. In 2020, a retrospective cohort study in China analyzed the influence of IADPSG criteria for diagnosing GDM on the perinatal outcomes and medical costs among GDM women and those with normal glucose tolerance (NGT) [48]. The results indicated that despite the rising costs of medical care, screening at 24–28 weeks in accordance with the IADPSG criteria with the one-step method can improve maternal and neonatal outcomes in the short term. These results are consistent with the HAPO study, which reported that there is a linear relation between hyperglycemia and adverse pregnancy risk outcomes, without an inflection point. Additionally, a study discovered that perinatal risks began to increase in women with glucose values that were regarded as “normal” [49].

In summary, mild GDM cases identified by the one-step method are associated with an increased risk of adverse pregnancy outcomes compared to women without GDM.

In comparison to other criteria, the IADPSG criteria would result in an elevated prevalence of GDM, which inevitably gives rise to a problem: the cost-effectiveness and financial implications of treating the “extra” women who are discovered by the IADPSG criteria. There are several cost-effective analysis cohort studies. The one-step method compared to the two-step would result in a greater burden on the system of medical healthcare. However, in terms of the outcomes of improving maternal and neonatal health, the one-step method is cost-saving and cost-effective [34,50,51]. Compared with the one-step method, the two-step method is likely to result in the omission of a considerable number of patients; approximately 25% of the patients are readily overlooked at the diagnostic stage. In 2012, Agarwal et al. [52] conducted an evaluation of the financial and operational implications of transitioning from the previous two-step GDM screening to one-step. The researchers discovered that transitioning to the one-step diagnostic method would result in a 42% rise in cost but a 36% decline in laboratory workload compared to the two-step method. Similarly, Duran et al. [35] reported that the application of the IADPSG criteria was cost-effective in 2014. A comparison of the C&C and IADPSG criteria indicated that the former may result in an unnecessary expenditure of EUR 14,358.06 per 100 women. They concluded that the one-step method would elevate treatment costs by EUR 3753.79. However, it would diminish laboratory expenses by EUR 1587.76, and further diminish delivery and neonatal intensive care center (NICU) costs of EUR 16,336.90. In a systematic review [53], Weile et al. searched cost-effectiveness studies published from 2002 to 2014 and discovered that cost-effectiveness ratios exhibited considerable variability. Most variation could be attributed to differences in geographic settings, diagnostic criteria, intervention approaches, and pregnancy outcomes.

For the diagnosis of GDM, several RCTs have been conducted to compare the one-step method applying IADPSG criteria with the two-step method employing C&C criteria. In 2018, Khalifeh et al. [54] reported the result of a small RCT which included 249 pregnant women. This study showed that the incidence of adverse pregnancy outcomes and the prevalence of GDM did not exhibit a statistically significant difference. However, the conclusion is not convincing enough, because the primary outcome was GDM incidence rate and not maternal or perinatal outcomes, which were the ultimate goals for choosing a proper screening method. A meta-analysis including three RCTs found that, compared to the two-step method, the one-step method could improve maternal and perinatal outcomes [55]. However, there are some limitations in these trials. To date, there are no published large-scale RCTs with enough significant evidence that use pregnancy outcomes as the primary endpoint to compare the one- and two-step methods in cost-effectiveness.

### 6.4. Universal Screening Versus Selective Screening

Currently, the debate over whether universal or selective screening strategies are preferable for diagnosing GDM remains unresolved. Both the OGTT and the 50 g GCT are time-consuming, unpleasant, and expensive for pregnant women. As a result, some professional associations advocate for selective screening based on risk factors to identify those at higher risk of GDM. The diagnostic OGTT is only applied to pregnant women at risk for GDM, which would lower the burden for pregnant women and the system of healthcare. The risk factors for GDM vary according to different criteria. The traditional risk factors include maternal age ≥ 35 years, body mass index (BMI) ≥ 25 or 30 kg/m^2^, history of GDM, bearing a child with macrosomia, and family history of diabetes. On the contrary, in universal screening, all pregnant women receive biochemical tests, including either a diagnostic OGTT or 1 h 50 g GCT followed by a diagnostic OGTT. According to the online survey organized by the EBCOG in 2015 [29], of all European associations recommending the IADPSG criteria, 52.6% recommended them based on risk factors, while 47.4% advocated for universal screening using either the one- or two-step method.

Several studies have compared the effectiveness of universal versus selective screening. A recent study [56] found that selective screening could miss approximately one-third of women with GDM who lacked risk factors but still experienced higher rates of adverse pregnancy outcomes than women without GDM. In an Italian retrospective cohort study, Pintaudi et al. [57] reported a GDM prevalence of 11.1% under a universal screening strategy. Of the total population, 58.3% had risk factors, and selective screening would miss 23% of GDM cases. These proportions were quite similar to those in another study [58], which reported that selective screening would miss one-sixth of women with GDM. According to the results of a cohort study in Paris, Cosson et al. [59] found that approximately one-third of women with GDM exhibited no discernible risk factors and were consequently overlooked through the method of selective screening. These women, despite lacking risk factors, had a higher incidence of adverse pregnancy outcomes than those without GDM. Therefore, Cosson et al. posited that universal screening could diminish the interval between the diagnosis and access to specified care for GDM. Therefore, the French guidelines rejected the proposal for selective screening. A number of researchers have concluded that the selective screening strategy avoided a small number of tests while substantially complicating the screening procedure [60]. For example, the American College of Obstetricians and Gynecologists (ACOG) [61] indicated that only 10% of the population could avoid OGTT according to risk factor-based screening. Thus, many physicians may opt to implement universal screening for pregnant women during clinical practice. Collectively, the current evidence deriving from a series of cohort studies tends to favor the use of universal screening. Many societies, such as the WHO, CDA, ACOG, and Australasian Diabetes in Pregnancy Society (ADIPS), have suggested using universal screening for the diagnosis of GDM [62,63,64].

### 6.5. Early Screening for Overt Diabetes and GDM

During the past few decades, the growing prevalence of advanced maternal age and obesity has contributed to an increased incidence of both pre-existing diabetes and GDM [65,66]. During early pregnancy, a significant proportion of women may have undiagnosed pre-existing diabetes or early-onset GDM. There is a higher risk of adverse pregnancy outcomes, and there are many benefits of early identification and intervention [67]. One goal of early testing is to recognize women at low or high risk for GDM. This risk classification could potentially reduce the necessity for universal diagnosis during the second trimester and further reduce the medical burden. Another significant objective is to detect women with pre-existing diabetes and initiate treatment as soon as possible. Various health organizations, including the ADA, ACOG, FIGO, and IADPSG, have reached a consensus that early screening for pre-existing overt diabetes is a beneficial practice during pregnancy, either for all pregnant women or for those with risk factors [68]. According to the ADA recommendation [20], an FPG ≥ 126 mg/dL (7.0 mmol/L), random plasma glucose (RPG) ≥ 200 mg/dL (11.1 mmol/L), 2 h plasma glucose during a 75 g OGTT ≥ 200 mg/dL (11.1 mmol/L), or glycated hemoglobin (HbA1c) ≥ 6.5% would constitute grounds for a diagnosis (see Table 3). An RPG of ≥200 mg/dL (11.1 mmol/L) should be used for diagnosis in a woman presenting with typical symptoms of hyperglycemia or a hyperglycemic crisis. Conversely, there was no uniformly applicable optimal method for the early screening of GDM. Many professionals discouraged screening for GDM in the initial trimester because of the lack of sufficient evidence to support the advantages of early diagnosis and treatment [67]. Despite this, various strategies have been proposed for early GDM detection. Diagnostic methods generally fall into two categories: direct and indirect. Direct methods include FPG and OGTT, while indirect methods encompass HbA1c testing. This section will explore the most commonly used approaches in detail.

In 2010, IADPSG recommended that GDM could be diagnosed based on FPG values between 92 and 125 mg/dL (between 5.1 and 6.9 mmol/L) at any stage during gestation (encompassing the initial trimester). However, this method was questioned because of the lack of sufficient supporting evidence [69]. A recent assessment of the FPG in the initial prenatal examination to diagnose GDM in China demonstrated that FPG values 110–125 mg/dL (6.1–6.9 mmol/L) were a significantly accurate predictor for the development of GDM in the population [69]. The thresholds were derived from the HAPO study, which measured FPG and OGTT glucose levels [7]. Nevertheless, research has demonstrated that FPG levels decrease during the course of pregnancy [70]. In 2013, a study conducted by Zhu and Yang revealed that over 60% of women with FPG levels exceeding 92 mg/dL (5.1 mmol/L) at the initial prenatal examination were not diagnosed with GDM at 24–28 weeks of gestation [69]. This implied that numerous women with elevated FPG levels in early pregnancy are not diagnosed with GDM during the second trimester. Owing to these reasons, IADPSG issued a statement in 2016 recommending the discontinuation of the FPG cut-off during early pregnancy [67]. Evidence supporting the use of first-trimester FPG alone to diagnose GDM remains insufficient, and current guidelines do not recommend screening or treatment of GDM before 24 weeks of gestation [20,71]. Nonetheless, elevated FPG levels can still be considered a significant risk factor for GDM. Previous studies have demonstrated a correlation between GDM diagnosed at 24–28 weeks of gestation and first-trimester FPG, utilizing the IADPSG thresholds. The concordance measures, represented by the area under the receiver operating characteristic curve (ROC-AUC), ranged from 0.614 to 0.654 [69,72].

An early 75 g OGTT during the initial trimester could be also utilized for overt diabetes diagnosis if the 2 h PG exceeds 200 mg/dL (11.1 mmol/L), following guidelines from FIGO and WHO [26]. In 2014, an RCT in Turkey assessed the diagnostic effectiveness of three methods utilized for GDM screening in the initial trimester: FPG, a two-step method (50 g GCT followed by 100 g OGTT using ADA or C&C criteria), and a one-step method (75 g OGTT using the IADPSG criteria) [73]. The results showed that GDM was diagnosed in 5.1%, 6.0%, and 11.3% of women using the FPG, one-step, and two-step methods, respectively. The FPG method demonstrated the least favorable performance, with an area under the ROC curve of 0.623, a 47.17% sensitivity, and a 77.37% specificity. The one-step method exhibited the most optimal performance, with an area under the ROC curve of 0.792, a sensitivity of 87.1%, and a specificity of 100%. In addition, the two-step method demonstrated superior performance with an area under the ROC curve of 0.708, a sensitivity of 68.18%, and a specificity of 100%. The findings suggested that the 75 g OGTT may be a more effective predictor for GDM than the two-step OGTT and the FPG test.

Several studies have been conducted to assess the efficacy of first-trimester HbA1c in detecting GDM. In 2017, Fong et al. [74] assessed early HbA1c values as an early predictor of progression to GDM in a cohort study. Their findings indicated that HbA1c levels of 5.7–6.4% were effective for recognizing women at high risk of developing GDM. Another multiethnic cohort study demonstrated that an early HbA1c ≥ 5.9% could effectively identify women at high risk of adverse pregnancy outcomes, independent of later GDM diagnosis [75]. In contrast, a cohort study including 1195 women in Spain found that the ROC-AUC for HbA1c in detecting GDM was 0.679. This study concluded that first-trimester HbA1c lacked adequate sensitivity and specificity to diagnose GDM [76]. However, employing both higher and lower thresholds could simplify the diagnostic process by decreasing the number of OGTTs. Similarly, a recent retrospective cohort study involving 2275 Indian women reported that HbA1c served as an independent predictor of GDM. Despite this, HbA1c alone lacked adequate sensitivity to serve as a standalone diagnostic tool for GDM [77].

## 7. Singleton Pregnancy Versus Twin Pregnancy

In general, women with twin pregnancies have a higher rate of GDM compared to those with singleton pregnancies. A 2009 study using multivariate regression analysis found that the risk of GDM in twin pregnancies was approximately double that of singleton pregnancies [78]. Similarly, a recent retrospective study comprising 270,843 women reported that women with twins exhibited a significantly higher risk compared to those with singleton pregnancies [79].

For diagnosis, the 50 g-GCT is currently employed for the screening of GDM in twin pregnancies, based on the same protocols utilized in singleton pregnancies [80]. However, there is a dearth of data concerning the accuracy of the 50 g-GCT for GDM in this population. The positive predictive value (PPV) of a 50 g-GCT for GDM in twin pregnancies when utilizing a threshold of 140 mg/dL (7.8 mmol/L) was approximately 23%, but there was a paucity of data regarding the sensitivity and specificity of the 50 g-GCT in twins [81], because in all available studies, OGTT was only performed if the 50 g-GCT was abnormal. Consequently, there is a dearth of data pertaining to the diagnosis of GDM in twins, prompting the Society for Maternal-Fetal-Medicine to advocate for further research in this area [82].

In terms of treatment and glucose management, there appears to be no significant difference between twin and singleton pregnancies. In 2022, Gupta et al. [83] found that the treatment strategies and insulin doses administered to GDM women with twin or singleton gestation did not differ significantly. It was recommended that treatment plans be designed to achieve glycemic goals, even in twin pregnancies. However, a study conducted in 2024 reached the conclusion that women with twin pregnancies and GDM treated with insulin may have a higher risk of preterm birth and excess uterine growth. Additionally, maternal age ≥ 35 years, overweight or obesity, and chronic hypertension were identified as significant risk factors for GDM during twin pregnancies [84]. These risk factors are the same as for singleton pregnancies.

In summary, the management of GDM in twin pregnancies remains a high-risk area requiring further investigation. Ongoing research aims to establish the most effective strategies for optimizing outcomes for both mothers and neonates.

## 8. Conclusions

This review provides a wide range of information on the diagnosis of GDM, including criteria and methods of diagnosis, and differences between universal and selective screening. It can serve as a reference point for the diagnostic criteria and methods of diagnosing GDM in diverse populations across different countries and regions.

To summarize, for the diagnosis of GDM, the selection of IADPSG criteria is optimal. The most widely adopted criteria for GDM are those established by the IADPSG. They can reduce insulin therapy in patients with GDM while increasing the rate of vaginal delivery, which is beneficial for the identification and treatment of patients with mild hyperglycemia. We can conclude and emphasize that the one-step method compared to the two-step method could improve pregnancy outcomes and reduce the costs (cost-effective) of diagnosing GDM. The method of universal screening is cost-effective compared to selective screening, which misses numerous pregnant women with GDM. In the end, early screening (before 14 weeks) for GDM in the first trimester has not been justified by the current studies. Pregnant women with twin pregnancies are at an elevated risk of developing GDM in comparison to those with singleton pregnancies.

It is highly recommended to use the IADPSG criteria, the one-step method, and universal screening for the diagnosis of GDM. Although the number of pregnant women with GDM will increase (due to the inclusion of many with mild GDM), the cost of clinical care for subsequent adverse pregnancy outcomes can be reduced.

## Figures and Tables

**Table 1:** Major criteria for the diagnosis of GDM.

	OGTT Type (g)	Steps	Abnormal Values (*n*)	Diagnostic Thresholds, mg/dL (mmol/L)				Reference
				FPG	1-h PG	2-h PG	3-h PG	
O’Sullivan 1964	100	2	≥2	90 (5.0)	165 (9.2)	145 (8.1)	125 (6.9)	[11]
NDDG 1979	100	2	≥2	105 (5.8)	190 (10.6)	165 (9.2)	145 (8.0)	[12]
C&C 1982	100	2	≥2	95 (5.3)	180 (10.0)	155 (8.6)	140 (7.8)	[13]
EASD 1996	75	1	≥1	108 (6.0)	-	162 (9.0)	-	[14]
WHO 1999	75	1	≥1	126 (7.0)	-	140 (7.8)	-	[15]
IADPSG 2010/WHO 2013	75	1	≥1	92 (5.1)	180 (10.0)	153 (8.5)	-	[16]
CDA 2013	75	2	≥1	95 (5.3)	190 (10.6)	162 (9.0)	-	[17]

GDM: gestational diabetes mellitus; OGTT: oral glucose tolerance test; FPG: fasting plasma glucose; PG: plasma glucose; NDDG: National Diabetes Data Group; C&C: Carpenter and Coustan; EASD: European Association for the Study of Diabetes; WHO: World Health Organization; IADPSG: International Association of Diabetes and Pregnancy Study Groups; CDA: Canadian Diabetes Association.

**Table 2:** One-step and two-step methods to diagnose GDM *.

	Criteria	Screening Method	Screening Thresholds, mg/dL (mmol/L)	Diagnostic Method	Diagnostic Thresholds, mg/dL (mmol/L)				Reference
			1-h PG		FPG	1-h PG	2-h PG	3-h PG	
One-step method Δ	IADPSG			75 g OGTT	92 (5.1)	180 (10.0)	153 (8.5)	-	[16]
Two-step method #	NDDG	50 g GCT	130 (7.2) or 140 (7.8)	100 g OGTT	105 (5.8)	190 (10.6)	165 (9.2)	145 (8.0)	[12]
	C&C				95 (5.3)	180 (10.0)	155 (8.6)	140 (7.8)	[13]

IADPSG: International Association of Diabetes and Pregnancy Study Groups; NDDG: National Diabetes Data Group; C&C: Carpenter and Coustan; FPG: fasting plasma glucose; PG: plasma glucose. OGTT: oral glucose tolerance test; GCT: glucose challenge test. * Adapted from American Diabetes Association. Δ “one-step method”: between 24 and 28 weeks of gestation, perform a 75 g OGTT, and measure plasma glucose levels at fasting, 1 h and 2 h. The OGTT needs to be performed the morning after an overnight fast for at least 8 h. GDM can be diagnosed if one of the three plasma glucose levels meets or exceeds the thresholds. # “two-step method”: between 24 and 28 weeks of gestation, perform a non-fasting 50 g GCT. If the 1 h plasma glucose ≥130 mg/dL (sensitivity is 90%) or ≥140 mg/dL (sensitivity is 80%), proceed to a 100 g OGTT and measure plasma glucose levels at fasting, 1 h, 2 h, and 3 h. GDM can be diagnosed when at least two of the four plasma glucose levels meet or exceed the thresholds.

**Table 3:** Diagnostic criteria of overt diabetes in pregnancy *.

Measure of Glycemia	Diagnostic Thresholds
FPG Δ	≥126 mg/dL (7.0 mmol/L)
HbA1c Δ	≥6.5% #
2 h plasma glucose during a 75 g OGTT Δ	≥200 mg/dL (11.1 mmol/L)
Random plasma glucose	≥200 mg/dL (11.1 mmol/L) **

FPG: fasting plasma glucose; HbA1c: glycated hemoglobin; OGTT: oral glucose tolerance test. * Adapted from the American Diabetes Association (ADA). Δ In the absence of unequivocal hyperglycemia, results need to be confirmed by repeated tests. # The test should be performed in a laboratory using a method that is NGSP-certified and standardized to the DCCT assay. ** In patients with classic symptoms of hyperglycemia or hyperglycemic crisis.

## Data Availability

No new data were created or analyzed in this study. Data sharing is not applicable to this article.
